# Breast Cancer and Exposure to Organochlorines in the CECILE Study: Associations with Plasma Levels Measured at the Time of Diagnosis and Estimated during Adolescence

**DOI:** 10.3390/ijerph16020271

**Published:** 2019-01-18

**Authors:** Delphine Bachelet, Marc-André Verner, Monica Neri, Émilie Cordina Duverger, Corinne Charlier, Patrick Arveux, Sami Haddad, Pascal Guénel

**Affiliations:** 1 Inserm U 1018, Center for research in Epidemiology and Population Health (CESP), University Paris-Sud, Paris-Saclay, 94807 Villejuif, France; delphine.bachelet@inserm.fr (D.B.); monicaneri2008@gmail.com (M.N.); emilie.cordina@inserm.fr (É.C.D.); 2 Department of Environmental and Occupational Health, School of Public Health, IRSPUM, Université de Montréal, C.P. 6128, Succursale Centre-Ville, Montréal, QC H3C 3J7, Canada; marc-andre.verner.1@umontreal.ca (M.-A.V.); sami.haddad@umontreal.ca (S.H.); 3 Department of Toxicology, University of Liège, Sart Tilman University Hospital, 4000 Liège, Belgium; c.charlier@chuliege.be; 4 Department of Medical Information, Centre Georges-François Leclerc, 21000 Dijon, France; parveux@cgfl.fr

**Keywords:** breast cancer, persistent organic pollutants, endocrine disruptor chemicals, physiologically based pharmacokinetic modeling, case-control study

## Abstract

Exposure to environmental chemicals with hormonal effects, such as organochlorine compounds (OCs), during developmental periods of breast cells may have an impact on the incidence of breast cancer later in life. However, the assessment of exposure to these chemicals that occurred in early life at the time of breast cancer development in adult women is a difficult challenge in epidemiological studies. Plasma levels of the OCs p,p’-dichlorodiphenyl dichloroethene (DDE) and polychlorinated biphenyl congener 153 (PCB153) were measured in 695 cases and 1055 controls of a population-based case-control study conducted in France (CECILE study). Based on these values, we used a physiologically-based pharmacokinetic (PBPK) model to estimate PCB153 levels at age 11–20 years when the women were adolescents. Overall, there was no clear association between breast cancer risk and measured levels of DDE and PCB153 at the time of diagnosis, but there was a trend of decreasing odds ratios of breast cancer with increasing DDE and PCB153 levels in women aged 50 years and over. The PBPK model revealed that PCB153 concentrations estimated during adolescence were highest in the youngest women born after 1960 who reached adolescence at a time when environmental contamination was maximum, and very low in the oldest women who attained adolescence before the contamination peak. Negative associations between breast cancer and PCB153 estimates during adolescence were also found. The negative associations between DDE and PCB153 levels measured at the time of diagnosis or estimated during adolescence in our study were unexplained. Further investigations are needed to clarify whether this finding is real or related to study artifacts. However, this study suggests that using PBPK models in epidemiological studies to back-estimate OC exposures during early life stages may be useful to address critical questions on cancer development.

## 1. Introduction

With approximately 1.7 million new cases each year around the world, breast cancer is the most frequent malignant disease among women [1]. It is estimated that over 54,000 women are diagnosed with breast cancer each year in France [2]. Besides well-established risk factors related to hormones and reproduction, environmental exposures may play a role in the rising incidence of the disease [3].

There has been increasing interest in the effects of synthetic chemicals with hormonal properties, or endocrine disrupting chemicals, on breast cancer risk. Among them, persistent organic pollutants are mostly lipophilic environmental pollutants that tend to accumulate and biomagnify in food chains, resulting in considerable exposure of living organisms. These compounds include polychlorinated biphenyls (PCBs), which were utilized worldwide in numerous industrial and commercial applications, and organochlorine pesticides like dichlorodiphenyltrichloroethane (DDT), which has been used in agriculture and as a pest control agent [4]. Both of these classes of organochlorine compounds (OC) have attracted attention because of their widespread occurrence in the environment and in human beings, and their potential ability to interfere with hormone-regulated processes [5]. In France and in most European countries, DDT was banned in the 1970s. Although a drastic reduction of DDT residues in human biological tissues has been observed, its most stable degradation product dichlorodiphenyldichloroethylene (DDE) still contaminates food of animal origin [6]. PCB industrial production ceased in 1987 in France. Due to the use and degradation of PCB-containing materials beyond that date, environmental pollution by PCBs has persisted, as, for example, in French rivers and in freshwater fishes [7]. PCBs and DDT have low genotoxic activity but they may contribute to breast cancer by altering mammary gland development [8] and promote tumor growth through hormonal pathways. It was shown in animal studies that exposure to endocrine disrupting chemicals might induce precocious development or delayed terminal end bud differentiation occurring around puberty [9]. The International Agency for Research on Cancer (IARC) has classified PCBs as carcinogenic to humans (Group 1), on the basis of sufficient evidence in humans and experimental animals [10]. The strongest evidence for risk to human health is for dioxin-like PCBs, though they cannot be considered as the only responsible agent of PCB carcinogenicity.

A review of the studies on DDT and DDE was recently conducted. The Working Group classified DDT as “probably carcinogenic to humans” (Group 2A) [11,12]. No clear association was found with breast cancer in more than 40 studies published since the 1990’s. However, the group of experts noted that DDT and DDE levels were almost invariably measured in blood or fat of adult women, and highlighted the possible importance of early-life exposure, which was not assessed in the epidemiological studies. Exposure to hormonally active chemicals during developmental windows of heightened susceptibility, such as *in utero* or puberty, may be crucial for the occurrence of breast cancer later in life. 

Assessing exposure during early life periods of adult women is a difficult challenge in breast cancer epidemiology, because the time interval between the relevant exposure period of interest and the disease occurrence spans several decades. This is an important issue, because exposures measured close to diagnosis (or generally a few years before in prospective cohort studies) may be relevant to tumor promotion (e.g., through estrogen- or progesterone-mediated pathways), but not to earlier stages of breast cancer development [3]. In a few instances, it has been possible to couple quantitative assessment of exposure during susceptibility life stages with follow-up of sufficient length (several decades). This was the case of three case-control studies nested in the same pregnancy cohort, whose participants had blood draws before and soon after delivery in the 1960’s. This unique design allowed researchers to demonstrate the association of some PCB congeners and DDT species with breast cancer in the mothers as well as in the daughters, after more than 50 years of follow-up [13,14,15].

In order to assess early exposure to PCB in a population where no long-term follow-up is available, our group tentatively used a physiologically-based pharmacokinetic (PBPK) model based on PCB concentrations measured in adult women. A PBPK model incorporates data on the absorption, distribution, metabolism, and excretion of chemicals, allowing the back-extrapolation of lifetime exposure profiles [16]. We developed a PBPK model based on plasma concentrations of PCB153 in breast cancer patients and age-matched controls measured at the time of breast cancer diagnosis to estimate PCB153 levels in earlier life periods, incorporating historical data on environmental contamination by PCBs, as well as individual information on weight, height, reproductive history and breast feeding, which are known predictors of PCB levels [17]. 

In the present paper, we investigated the association between breast cancer and plasma levels of DDE and PCB153 measured at the time of breast cancer diagnosis in a large case-control study conducted in France. We also estimated PCB153 concentration during adolescence, using a PBPK model, and tentatively explored its association with breast cancer.

## 2. Methods

### 2.1. Recruitment of Cases and Controls 

We conducted a population-based case-control study on breast cancer in *Côte d’Or* and *Ille-et-Vilaine*, two administrative areas (*départements*) located in Eastern and Western part of France, respectively.

Cases were breast cancer patients below 75 years of age with invasive or in situ breast carcinoma diagnosed from April 2005 to March 2007, identified from the main cancer treatment centers in each area and from smaller public and private hospitals. The objective was to include all incident cases resident in the study areas during the study period. Among the 1556 eligible cases identified, 163 refused to participate, 151 could not be contacted and 7 died before the interview, leaving 1235 cases included in the study. Among them, 1080 (87%) accepted to have a blood draw. For 385 of these cases, the blood was drawn after a first chemotherapy treatment. To avoid potential influence of chemotherapy on OC measurements, these women were excluded from the statistical analyses, leading to 695 cases included in the present study.

Population controls were selected among women residing in the study areas at the time of the case diagnosis and were frequency-matched to the cases by 10-year age group. They were recruited for the study during the same period as the cases in 2005–2007. Quotas by socio-economic status (SES) were set a priori to control for potential selection bias arising from differential participation rates across SES categories [18]. We aimed to obtain a distribution by SES category in the control group identical to the SES distribution in the general population of women, conditionally to age.

To recruit the controls, phone numbers of private homes were selected at random from the telephone directory of each study area. Unlisted numbers were also made available. When a woman living in the residence was reached by phone, she was invited to participate to the study as long as the predefined number of women in her age and SES stratum was not reached. Among the 1731 eligible controls identified by phone, 1317 (76%) accepted to participate to an in-person interview, and 1055 (80%) accepted to have a blood draw and were included in the present study.

All subjects signed informed consent before being included in the study. 

The study was approved under the identification code 04-53 by the French Ethic Committee (Comité consultatif de protection des personnes dans la recherche biomédicale (CCPPRB) Kremlin-Bicêtre) in January 2005. 

### 2.2. Data and Sample Collection 

A structured questionnaire was used during in-person interviews by trained nurses to obtain information on socio-demographic characteristics, menstrual and reproductive history, height, weight by decade of adult life starting at age 20, personal medical history, family history of cancer, history of residence, lifetime occupational history and dietary habits. A blood sample (30 mL) was taken after the interview. Blood draws were aliquoted and stored at −80 °C until analysis.

### 2.3. Organochlorine Quantification

Levels of OCs were measured in the toxicology laboratory of the Sart-Tilman University Hospital in Liège, Belgium, in 2 mL plasma samples, using a gas chromatograph coupled to an ion trap mass spectrometer detector, as already described [17]. Briefly, sample preparation included a liquid-liquid extraction followed by a solid phase extraction (Bond Elut Certify). The eluate was evaporated to dryness, reconstituted, and then injected into the gas chromatograph. All solvents were pesticide grade quality. Reference standards of all chemicals were obtained from Cambridge Isotope Laboratories (Andover, MA, USA). Measurements were made for o,p’-DDT (limit of detection in µg/L (LOD): 0.12), p,p’-DDT (LOD: 0.64), o,p’-DDE (LOD: 0.03), p,p’-DDE (LOD: 0.51), hexachlorobenzene (HCB) (LOD: 0.39), lindane (LOD:0.48), heptachlor (LOD: 0.52), heptachlor epoxyde (LOD: 0.88), dieldrin (LOD: 0.36) and PCB 28 (LOD: 0.38), PCB52 (LOD: 0.07), PCB101 (LOD: 0.07), PCB118 (LOD: 0.45), PCB138 (LOD: 0.66), PCB153 (LOD: 0.50) and PCB180 (LOD: 0.72). We restricted subsequent analyses to the chemicals that were detectable at least in 50% of the subjects: p,p’-DDE and PCB153 (Table 1).

Concentration of triglycerides and cholesterol was also measured in the plasma samples. Total lipids in µg/L were calculated as triglycerides (µg/L) + 2.27 × cholesterol (µg/L) + 0.623 [19]. All concentrations of OC compounds were expressed in ng/g lipid.

### 2.4. PCB153 Lifetime Toxicokinetic Profiles

PCB153 was the PCB congener most frequently detected and was chosen for back-extrapolation using the PBPK model. Individualized lifetime PCB153 toxicokinetic profiles were simulated using the PBPK modeling framework developed by Verner et al. [16,20], carried out with acslX (Aegis Technologies Group, Inc., Huntsville, AL, USA). This model enabled the estimation of PCB levels in nine human tissue compartments: fetus, placenta, uterine tissue, brain, adipose tissue, richly perfused tissue, slowly perfused tissue, liver and mammary tissue, as well as excretion through metabolism and breastfeeding. Many of these pharmacokinetic processes are dependent on several physiologic parameters such as the volume and composition of organs, and the blood perfusion. The mathematical functions describing these processes are derived from population data.

The model incorporated the plasma level of PCB153 measured at the time of diagnosis for the cases and at the time of the interview for the matched controls, which will be referred to hereafter as the ’reference date’. For women with undetectable PCB153 (40%), we imputed random values below the LOD. To do this, we first categorized women with detectable PCB153 by age and weight gain during the last 10 years, which were the strongest predictors of PCB153 levels in our data [17]. Then, as PCB153 values detected in each of the four categories defined by age (50–60 years and ≥60 years) and body mass index (BMI) gain (<0.8 kg/m² and ≥0.8 kg/m²) followed a lognormal distribution, random values below the LOD were generated from the lognormal distribution function in each group and were attributed to the subjects (see Appendix A for details). 

The model also incorporated information on PCB153 determinants available from the study questionnaire: date of birth, age, height and weight by decade of life, age at pregnancies, and date of start and end of each breastfeeding period. 

Finally, the model incorporated each subject’s daily dose of PCB153, which was estimated based on environmental and food contamination history from 1930 on, as described by Verner et al. [20] (see Appendix B). Historical PCB production data were used for the period from 1930 (when PCB production started) to the 1970’s, when peak production occurred [21], while after 1977, data on the PCB content in food and dietary intake patterns in a European population were used [22]. Overall, contamination by PCBs was time-dependent with a sharp increase starting in the 1950’s, followed by a peak from 1970 to 1977 and a decrease from 1980 onwards. Subject-specific daily dose was calibrated so as to follow the profile of environmental contamination and to obtain a simulated PCB153 level at the reference date that matched the measured plasma level.

**Figure 1:**
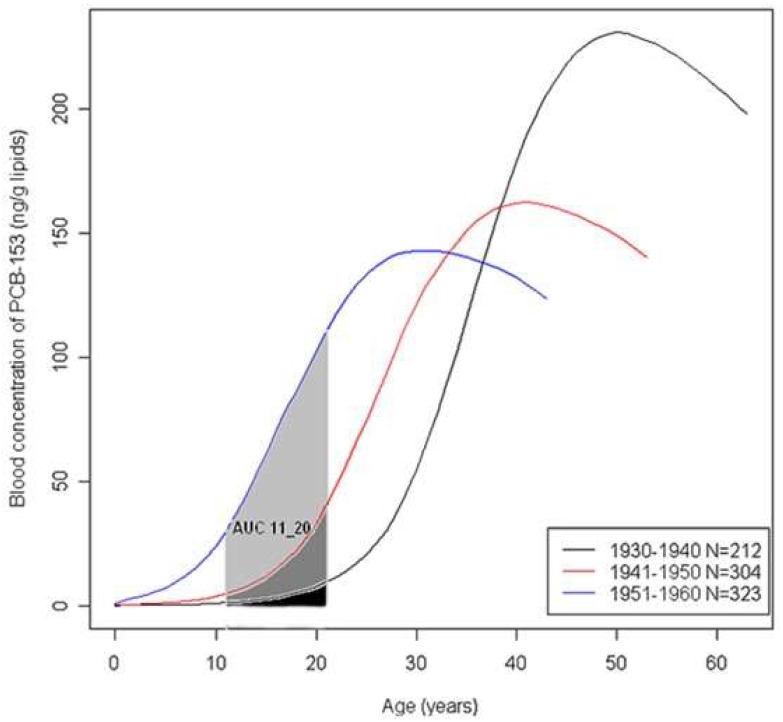
Mean lifetime toxicokinetic profiles according to the birth cohort. AUC 11_20 is the area under the PBPK-derived PCB153 concentration curve for the age period 11 to 20 years.

We extracted plasma concentrations from estimated exposure profiles from 11 years of age to age at the reference date. Earlier periods of life were not considered as exposure during infancy is largely dependent on breastfeeding by the mother, which was not known in our study. In the present paper we focused on adolescence, which we broadly defined as the period between 11 and 20 years of age. Therefore, internal levels of PCB153 were calculated from the individual lifetime toxicokinetic profiles as the area under the curve (AUC) of the lipid-adjusted blood concentration in ng/g lipid in the age period 11–20 years (Figure 1).

Although it has been suggested that breastfeeding duration can influence PCB levels through adolescence [23,24], the contribution of other factors and exposure routes becomes more important as the child ages [23].

### 2.5. Statistical Analyses 

In the analyses based on the DDE and PCB153 measurement at reference date, plasma levels of PCB153 (μg/l) were divided in four classes. The LOD was used as a natural cut-off point to define the lower exposure category, and tertiles of the PCB153 distribution among controls with values above the LOD were used to define the three upper categories. We also conducted stratified analyses by age group, used as a proxy for menopausal status (<50 years; ≥50 years).

In all analyses, odds ratios (OR) and 95% confidence intervals (CI) were estimated using unconditional logistic regression adjusting for age (5-year age groups), study area reference date, education, parity, age at first full-term pregnancy, body mass index, hormone replacement therapy, family history of breast cancer, history of benign breast disease, breastfeeding, and date of interview. We included these variables in the multivariate model as they are well established breast cancer risk factors or as we found them to be significantly associated with the risk of breast cancer in univariate analyses. Missing covariate data were modeled using missing value indicator categories. Tests for trends were performed by modeling categorical exposures as ordinal variables after assigning median values to each exposure category. Polytomous logistic regression models were used to conduct analyses by receptor status, namely, for estrogen receptor (ER+/ER−), progesterone receptor (PR+/PR−) and human epidermal growth factor receptor 2 HER2 (HER2+/HER2−) and by diagnostic status (in situ and invasive).

In the analyses of PCB153 at adolescence, exposure was categorized according to the tertiles of PBPK-derives estimates of PCB153 distribution among controls. Because these estimates varied considerably by birth cohort, the analysis was made separately for women born in 1930–1940, 1941–1950, 1951–1960 and 1961–1981.

Analyses were performed using R software (version 3.3.1. R Core Team (2018). R: A language and environment for statistical computing. R Foundation for Statistical Computing, Vienna, Austria. URL https://www.R-project.org/); all tests were two-sided, and *p* < 0.05 was considered statistically significant.

## 3. Results

The distribution of cases and controls by age, study area and well-established risk factors for breast cancer is shown in Table 2.

Breast cancer risk increased with earlier age at menarche, low parity, low BMI in women before menopause, late age at first full-term pregnancy, current use of menopausal hormone therapy, family history of breast cancer, and history of benign breast disease. Breast cancer incidence also increased with shorter duration of breastfeeding but the association did not reach statistical significance. No association with BMI was apparent among post-menopausal women.

Plasma DDE levels were not associated with breast cancer risk, the adjusted OR being 0.93 (95% CI 0.73, 1.18) for women in the highest exposure group compared to women with levels below the LOD (Table 3). A statistically significant negative association was found with the intermediate level of exposure, although the trend was not significant. After stratification by age, no association was shown among women who were less than 50 years old, while a negative association was apparent among the oldest subjects for the two highest levels of exposure (OR 0.63; 95% CI 0.47, 0.85 for the intermediate level and OR 0.81; 95% CI 0.61, 1.07 for the highest level), with a borderline statistically significant dose-response trend (*p*-trend 0.06). 

Adjusted OR was 0.75 (95% CI 0.57, 0.97) for women in the highest category of PCB153 concentration compared with those with levels below the LOD (Table 3). In analyses stratified by age, a similar negative association was only observed in women aged 50 years or older (OR = 0.65; 95% CI 0.48; 0.89, *p*-trend 0.09).

Results were essentially unchanged after stratification by hormonal receptor status (ER, PR) and by in situ (*n* = 123) or invasive (*n* = 572) breast cancer (data not shown).

Exposures to PCB153 at adolescence (11–20 years) estimated with the PBPK model varied considerably depending on the calendar year period the women reached adolescence. Back-extrapolated levels during adolescence increased sharply from the oldest birth cohort (born 1930–1940, calendar years of adolescence 1941–1960) to the youngest birth cohort (born 1951–1960, calendar years of adolescence 1962–1980) (Figure 1). The age gradients of PCB153 levels estimated at puberty and measured at the time of cancer diagnosis were therefore in the opposite direction. 

Odds ratios associated with PBPK-derived estimates of PCB153 exposure during adolescence were calculated after stratification by birth cohort (Table 4). Analysis of estimates for all women combined was not applicable because there was little overlap between the exposure distributions by birth cohort. The odds ratio for breast cancer decreased with PCB153 level during adolescence among women born in 1930–1940 (OR = 0.24, 95% CI 0.13, 0.42 in the highest exposure tertile), in 1941–1950 (OR = 0.53, 95% CI 0.32, 0.85) and in 1951–1960 (OR = 0.38, 95% CI 0.24, 0.60). In addition, we conducted sensitivity analyses excluding women with PCB153 below the LOD, but results were essentially unchanged and are not shown.

## 4. Discussion

Overall, plasma levels of DDE and PCB153 measured at the time of breast cancer diagnosis were not clearly associated with risk in our study, but a negative association between concentration of both OCs and breast cancer risk was seen in women above age 50 years. We also estimated PCB153 in the age span 11–20 years using a PBPK model and showed that contamination by PCB153 during adolescence was very low among women born in 1930–1940 and increased several order of magnitude among women born in 1961–1980. We found that PCB153 levels estimated during adolescence were also negatively associated with breast cancer risk, regardless of the birth cohort.

It has long been suggested that, due to their hormonal effects, OCs could be causally associated with breast cancer risk [3]. DDT was classified by IARC as a probable carcinogen for liver and non-Hodgkin lymphoma (Group 2A) [11], but mixed and inconclusive results were found in studies on breast cancer, with no overall association in meta-analyses [25,26]. PCBs were classified by IARC as Group 1 carcinogens for skin melanoma [10]; the IARC group of experts also noted that PCBs may play a role in breast cancer, but it admitted that study results were very heterogeneous and that chance findings could not be excluded [10]. In addition, recent meta-analysis found no association between breast cancer and PCB153 levels in plasma or in adipose tissue [27]. Mixed and inconclusive results were also noted by Rodgers et al. in their systematic literature review [3], but ORs for breast cancer associated with high exposure to PCB153 were significantly increased in a recent study of Inuit women [28].

Mammary gland cells are more vulnerable to genotoxic damage from environmental carcinogens during periods of cell growth and differentiation, e.g. during life *in utero*, adolescence and pregnancy, and early exposures may alter breast development and increase adult susceptibility to breast cancer [3]. In most studies on OC exposure and breast cancer risk, DDE and/or PCBs were measured in the blood or adipose tissues either at the time of cancer diagnosis in case-control studies, or typically a few years before diagnosis in prospective cohort investigations, without consideration of critical exposure windows during lifetime. The lack of exposure measures during etiologically relevant periods of breast cancer development may thus explain the null or inconclusive results for the association between breast cancer risk and OC exposure in epidemiological studies. 

A notable exception is the study by Cohn et al. that reported on a prospective cohort of young pregnant women (CHSD Child Health and Development Study) who provided blood samples when giving birth in 1959–1967, and who were followed-up for breast cancer incidence during a median time of 17 years. High levels of serum p,p´-DDT predicted a statistically significant 5-fold increased risk of breast cancer among women who were under 14 years of age in 1945, when DDT came into widespread use [14]. Follow-up during 52 years of the 9300 female offspring born from these women showed that o,p’-DDT measured in the maternal serum predicted the daughters’ breast cancer risk [13]. In the same cohort, the authors reported that the concentration of PCB203 in the archived serum samples was associated with an increased incidence of breast cancer before age 50 in 112 case-control pairs, while PCB167 and PCB187 were associated with a decreased risk. No association with PCB153 was observed [15]. The findings suggest that exposure to DDT or PCBs during periods of breast cancer development with increased susceptibility to carcinogens may have an impact on the risk of breast cancer later in life. 

### 4.1. PBPK Model for Critical Exposure Windows

The CHSD cohort is unique in that it is based on archived serum samples collected from young women at a time when environmental contamination by OCs was elevated, and on extensive follow-up to study breast cancer incidence. Because to our knowledge, no other similar cohort is available to explore breast cancer risk in relation to early exposure to OCs, we used pharmacokinetic modelling as an exploratory approach to estimate PCB153 exposure at adolescence in the women of our case-control study. PCB153 was chosen as it has a long biological half-life, and as it may be seen as a marker of exposure to other OCs [29]. We have decided not to explore exposure before age 10 because data on how the woman was breastfed are important predictors of OC levels and were not available in our study. The PBPK-estimated PCB153 levels during adolescence varied considerably among women depending on the calendar years they reached that age. Back-extrapolated levels were lowest in women born in 1930–1940 who reached adolescence at a time when PCB use was not widespread, and were highest in women born in 1961–1981 who reached adolescence during the peak of PCB use before it was banned. These exposure estimates contrasted with measured levels at the time of breast cancer diagnosis, that were highest in older women who bio-accumulated PCBs in body tissues for a longer life span period. Because of strong PCB exposure gradients, we conducted analyses stratified by birth cohort. 

The negative association with breast cancer observed for PCB153 concentrations at the time of cancer diagnosis in women aged 50 years and over was also observed for PCB153 estimated during adolescence in women born before 1961. Negative associations with breast cancer have been reported in several studies for total PCBs [30] or for specific PCB congeners, including PCB153 [14,31,32,33]. A marked negative association between levels of PCBs, including PCB153, and risk of testicular germ cell tumors was observed in a study among young men using pre-diagnostic blood samples [34]. Similarly, in a case-control study in Guadeloupe (French West Indies), PCB153 concentration in plasma collected prior to any treatment was negatively associated with prostate cancer [35]. Several hypotheses can be put forward to explain these findings. First, PCB153 might actually decrease the risk of breast cancer though its anti-estrogenic activity corroborated by experimental studies showing that PCB153 induces estrogen-metabolizing cytochrome P450 enzymes and reduces estradiol levels [36,37,38]. Second, because PCB153 is a very long half-life PCB congener [29], the negative association with breast cancer may result from collinearity with chemicals having known anti-estrogenic effects such as dioxins or dioxin-like PCB congeners [39,40]. Third, reverse causality is a possible explanation for the negative association. For example, OC levels measured in the blood samples collected in breast cancer patients after diagnosis may have been affected by recent weight loss [41] or other factors associated with the development of the tumor that can ultimately result in decreased OC concentrations in breast cancer patients. Fourth, we cannot exclude a selection bias during the recruitment phase of study subjects. For example, exposure to PCBs of the controls could be biased (e.g., be too high) if the selection of controls by place of residence was not fully balanced with that of the entire population in the study areas. However, PCB153 exposure levels among controls in our study (median 106.3 ng/g lipids in women aged 50 years and over) were close to PCB concentrations observed in a survey among French women conducted in the same period [42]. No clear explanation is currently available for the negative associations observed in our study, but this finding, as well as similar observations in previous studies, should be carefully scrutinized. 

### 4.2. Strengths and Limitations

Our study was designed to minimize potential for selection bias of both cases and controls: we attempted to include all incident cases diagnosed during the study period among women living within well-defined geographical areas, and included age-matched population controls who were representative of the source population by socioeconomic status. 

Several sources of imprecision may have affected laboratory measurements of OC concentrations and estimates of PCB153 exposure at adolescence. However, exposure was assessed blindly as for the case-control status, and exposure misclassification due to laboratory measurements or inaccuracies in PBPK modeling was non-differential, so it is not likely to account for the observed associations. The limits of detection of OC in plasma samples were relatively high in our study. However, PCB153 and DDE used in the analysis were detected in the majority of women and values below the LOD were imputed as described in Appendix A.

PBPK model estimations heavily rely on measured plasma levels at the time of diagnosis and on the daily oral dose of PCB estimated from the 1940s to the date of recruitment in the study using environmental contamination profile derived from literature data (see Appendix B). Because the same average daily oral dose was applied to each woman, without consideration of inter-individual variations in exposure, the PCB153 estimate during adolescence should be considered indicative and may be subject to large fluctuations. Nevertheless, PBPK models may help to better capture early-life exposures to OCs and may be useful for assessing to circumvent the limitations of traditional approaches [20]. 

## 5. Conclusions

We have shown that breast cancer risk was negatively associated with plasma concentrations of DDE and PCB153 measured at the time of cancer diagnosis in adult women and estimated during adolescence. This negative association was unexplained but it follows similar results in previous studies on OC exposure in hormone-dependent cancers. These findings should be carefully scrutinized to determine whether they are real, or due to study artifacts. The PBPK models were used as an exploratory approach to back-estimate PCB exposures during adolescence. This approach may be useful for assessing exposure to OC in early life periods and should be further developed to address critical questions on cancer etiology in observational studies. 

## Figures and Tables

**Table 1:** Percentage of detection and plasma concentration distribution of organochlorine pollutants (ng/g lipids) among women participating in the CECILE study, stratified by age.

Organochlorine ^a^	Detection Frequency (%)	25th Percentile	50th Percentile	75th Percentile	90th Percentile	Max
**Controls**						
**<50 years (*N* = 357)**						
p.p’-DDE	38.1	<LOD	<LOD	131.5	209.1	839.6
PCB138	7.0	<LOD	<LOD	<LOD	<LOD	230.7
PCB153	36.1	<LOD	<LOD	103.1	165.1	496.8
PCB180	12.6	<LOD	<LOD	<LOD	131.7	353.0
**≥50 years (*N* = 698)**						
p.p’-DDE	61.7	<LOD	105.7	196.0	342.3	2440.0
PCB138	26.1	<LOD	<LOD	79.1	155.0	748.0
PCB153	70.9	<LOD	106.2	161.7	239.4	1218.0
PCB180	27.8	<LOD	<LOD	100.0	170.8	654.2
Cases						
**<50 years (*N* = 162)**						
p.p’-DDE	40.1	<LOD	<LOD	141.3	288.0	2051.0
PCB138	11.1	<LOD	<LOD	<LOD	113.0	224.5
PCB153	42.0	<LOD	<LOD	110.6	210.3	440.8
PCB180	15.4	<LOD	<LOD	<LOD	177.8	384.2
**≥50 years (*N* = 533)**						
p.p’-DDE	58.2	<LOD	93.7	201.4	374.2	2196.0
PCB138	21.6	<LOD	<LOD	<LOD	140.3	756.2
PCB153	66.2	<LOD	94.7	146.2	205.4	559.0
PCB180	23.3	<LOD	<LOD	<LOD	158.0	798.1

^a^ o,p’-DDE, Lindane, Heptachlor, Heptachlor-epoxyde and PCB congeners 28, 52 and 101 were below the LOD in all cases and controls. o,p’-DDT and p,p’-DDT were detected in one and two cases respectively. Hexachlorobenzene was detected in six cases and four controls. PCB 118 was detected in three cases and four controls; Distributions of these compounds among controls as previously published [17]; DDE: dichlorodiphenyldichloroethene, DDT: dichlorodiphenyltrichloroethane, LOD: limit of detection, PCB: polychlorinated biphenyls.

**Table 2:** Distribution of cases and controls according to age, study area and breast cancer risk factors.

Matching Variables and Breast Cancer Risk Factors		Cases (*n* = 695)		Controls (*n* = 1055)		OR ^a^	95% CI
		*n*	%	*n*	%		
Age	<40	28	4	114	11		
(Years)	40–49	134	19	243	23		
	50–59	245	35	318	30		
	≥60	288	41	380	36		
Study Area	Côte d’Or	210	30	368	35		
	Ille-et-Vilaine	485	70	687	65		
Age at menarche	<14	434	63	652	62	1.00	reference
(Years)	14	131	19	204	20	0.91	[0.74, 1.13]
	15+	121	18	189	18	0.89	[0.71, 1.11]
	missing	9		10			
Parity	0	76	11	72	7	1.00	reference
	1	106	15	134	13	0.76	[0.53, 1.08]
	2	281	40	373	35	0.69	[0.51, 0.94]
	3	167	24	324	31	0.45	[0.32, 0.62]
	4+	65	9	152	14	0.34	[0.23, 0.50]
Age at first full term	<22	152	25	284	29	0.80	[0.64, 1.01]
Pregnancy among parous	22–24	189	31	297	30	1.00	reference
Women (years)	25–27	132	21	235	24	0.92	[0.72, 1.17]
	28+	146	24	167	17	1.48	[1.15, 1.90]
Duration of breastfeeding	never breastfed	301	49	458	47	1.00	reference
Among parous women	<26	224	37	370	38	1.00	[0.82, 1.21]
(Weeks)	26–52	56	9	95	10	1.00	[0.73, 1.36]
	53+	31	5	57	6	0.88	[0.59, 1.30]
	missing	7		3			
Body mass index	*<50 years*						
(kg/m²)	<18.5	11	7	8	2	2.61	[1.16, 6.06]
	18.5–24.9	118	73	231	65	1.00	reference
	25–34.9	24	15	80	22	0.56	[0.36, 0.86]
	35+	9	6	37	10	0.46	[0.23, 0.86]
	missing			1			
	*≥50 years*						
	<18.5	14	3	16	2	1.12	[0.6, 2.1]
	18.5–24.9	284	54	358	51	1.00	reference
	25–34.9	153	29	217	31	0.89	[0.71, 1.11]
	35+	78	15	106	15	0.92	[0.69, 1.22]
	missing	4		1			
Hormonal replacement therapy (current intake)	No	570	87	904	90	1.00	reference
	Yes	85	13	96	10	1.31	[1.00, 1.72]
	missing	40		55			
Family history of breast							
Cancer in first-degree	No	574	83	953	90	1.00	reference
Relatives	Yes	121	17	102	10	1.93	[1.51, 2.45]
Benign breast disease	No	404	58	743	70	1.00	reference
	Yes	290	42	311	29	1.62	[1.37, 1.93]
	missing	1		1			

^a^ Odds ratios adjusted for age and study area; CI: confidence interval.

**Table 3:** Odds ratios of breast cancer for plasma DDE and PCB153 concentrations measured at diagnosis for the cases and the matched controls of the CECILE study.

		Cases	Controls	OR ^a^	95% CI	*p*-Trend
	**DDE (ng/g lipids)**					
**All**	<LOD	310	480	1.00	reference	0.11
	51.3–131.5	125	186	0.97	[0.76, 1.24]	
	131.5–212.4	91	185	0.64	[0.49, 0.83]	
	≥212.4	150	189	0.93	[0.73, 1.18]	
**<50 years**	<LOD	96	215	1.00	reference	0.85
	53.1–130.6	18	45	0.75	[0.42, 1.30]	
	130.6–181.1	15	45	0.71	[0.39, 1.25]	
	≥181.1	32	46	1.48	[0.90, 2.41]	
**≥50 years**	<LOD	214	265	1.00	reference	0.06
	51.3–132.8	107	141	0.97	[0.73, 1.28]	
	132.8–222.2	80	140	0.63	[0.47, 0.85]	
	≥222.2	114	143	0.81	[0.61, 1.07]	
	**PCB153 (ng/g lipids)**					
**All**	<LOD	265	424	1.00	reference	0.28
	36.6–110.0	156	202	1.14	[0.90, 1.46]	
	110.0–162.6	133	204	0.89	[0.69, 1.14]	
	≥162.6	122	210	0.75	[0.57, 0.97]	
**<50 years**	<LOD	93	223	1.00	reference	0.32
	59.4–110.1	27	42	1.72	[1.01, 2.90]	
	110.1–155.4	13	42	0.65	[0.33, 1.22]	
	≥155.4	28	44	1.46	[0.85, 2.49]	
**≥50 years**	<LOD	172	201	1.00	reference	0.09
	36.6–110.0	129	161	1.00	[0.76, 1.32]	
	110.0–163.4	116	161	0.85	[0.64, 1.13]	
	≥163.4	98	166	0.65	[0.48, 0.89]	

^a^ ORs adjusted for age, study area, level of education, age at menarche, parity, age at first full-term pregnancy, body mass index, hormone replacement therapy, familial history of breast cancer, history of benign breast disease, breastfeeding, reference date; LOD: limit of detection.

**Table 4:** Associations between breast cancer and PCB153 circulating levels measured at the date of diagnosis for the cases and the matched controls or PBPK-derived estimates of PCB153 concentrations at age 11–20 years, stratified by birth cohort.

Birth Cohort	PCB153 at Diagnosis (ng/g lipids)	Cases	Controls	OR ^a^	95% CI	Calendar Years When Aged 11–20 Years	PCB153 at Age 11–20 Years ^b^(ng/g lipids)	Cases	Controls	OR ^a^	95% CI
1930–1940	<98.1	70	69	1.00	reference	1941–1960	<1.5	88	69	1.00	reference
	98.1–173.7	69	68	1.14	[0.73, 1.75]		1.5–2.8	59	68	0.51	[0.32, 0.82]
	≥173.7	41	70	0.58	[0.35, 0.89]		≥2.8	33	70	0.24	[0.13, 0.42]
1941–1950	<LOD	73	102	1.00	reference	1952–1970	<6.4	71	99	1.00	reference
	LOD–134.2	79	99	1.34	[0.91, 1.97]		6.4–12.5	63	98	0.65	[0.42, 0.99]
	≥134.2	51	100	0.62	[0.40, 0.94]		≥12.5	69	104	0.53	[0.32, 0.85]
1951–1960	<LOD	103	138	1.00	reference	1962–1980	<35.0	94	107	1.00	reference
	LOD–124.9	62	92	0.99	[0.67, 1.46]		35.0–63.0	69	106	0.67	[0.45, 0.99]
	≥124.9	48	93	0.52	[0.34, 0.79]		≥63.0	50	110	0.38	[0.24, 0.60]
1961–1981	<LOD	51	150	1.00	reference	1972–1990	<47.1	29	70	1.00	reference
	LOD–127.7	11	29	1.13	[0.52, 2.41]		47.1–83.6	23	66	0.73	[0.39, 1.36]
	≥127.7	18	30	1.15	[0.56, 2.30]		≥83.6	28	73	0.71	[0.38, 1.34]

^a^ OR adjusted for age, study area, level of education, age at menarche, parity, age at first full-term pregnancy, body mass index, hormone replacement therapy, family history of breast cancer, history of benign breast disease, breastfeeding, date of blood draw; ^b^ PCB153 at age 11–20 years was calculated from the AUC of the toxicokinetic profile in ng·h/g lipids divided by time between 11 and 20 years in hours to obtain ng/g lipids; LOD: limit of detection.

## Supplementary Materials

The following are available online at http://www.mdpi.com/1660-4601/16/2/271/s1, Figure S1: Temporal trend of environmental contamination levels reported as fractions of peak levels based on production data and estimated daily intakes.

## Appendix A. Imputation of Values below the Limit of Detection (LOD)

In each group, we estimated the distribution mean *µ* and variance *σ* from the quantiles.

Let *Y* be the variable corresponding to the logarithm of the PCB153 concentration; *Y* is a Gaussian variable.

Let *Y** = *Y* | *Y* < *s* where *s* is the logarithm of the LOD of *Y*. *Y** is the non-observed part of *Y*.

Let *Y^+^* = *Y* | *Y* ≥ *s*. So *Y^+^* is the observed part of *Y*.

Let *F* and *F** be the respective repartition functions of *Y* and *Y**.

For *y** < *s*, we have *F**(*y**) = *P*(*Y** ≤ *y**)

*F**(*y**) = *P*[(*Y*|*Y* < *s*) ≤ *y**]

Therefore: $F*(y*)=\frac{P(Y\leq y*,Y<s)}{P(Y<s)}$

$$F*(y*)=\frac{P(Y\leq y*)}{F(s)}$$

Let Φ be the repartition function of the standard normal distribution.

We have

(A1) $$F*(y*)=\frac{\Phi (\frac{y*-\mu }{\sigma })}{\Phi (\frac{s-\mu }{\sigma })}$$

The objective is to randomly simulate *y** according to the repartition function *F** determined from Equation (A1). To do this, we determined the reciprocal function *F**^−1^ from *F** by:

$$F*(y*)=\frac{\Phi (\frac{y*-\mu }{\sigma })}{\Phi (\frac{s-\mu }{\sigma })}=p\in \left[0,1\right]$$

$$F{*}^{-1}(p)=\sigma \times \Phi^{-1}(p\times \Phi (\frac{s-u}{\sigma }))+\mu =y*\in \left[0,s\right]$$

if *p* is the realization of a variable distributed according the uniform distribution on the interval [0,1], then $y*=\sigma \times \Phi^{-1}(p\times \Phi (\frac{s-\mu }{\sigma }))+\mu$ is the realization of a random variable distributed from *F**.

## Appendix B. The Daily Oral Dose

The temporal trend of the environmental contamination was estimated from production data [21] and European daily intake estimations [22] which were the most pertinent data found. We assumed a peak period from 1970 to 1977. These data were standardized by a division by the maximum reached for each factor (production and daily intake estimation) to make them range between 0 and 1. The environmental contamination curve was defined as a time-dependent function *F* between 0 and 1, differing according to the considered period. *F* equals 1 over the period 1970–1977 corresponding to the peak of the environmental contamination. *F* is an exponential function increasing for the period 1930–1970 and decreasing for the period 1977–2007, and was estimated to adjust itself to the standardized data of production and of daily intake from food by the method of least squares (see Figure S1).

The daily oral dose for time *t* corresponds to the multiplication of the function *F(t)* ranging between 0 and 1 by a maximal oral dose imposed in the model. This maximal oral dose is calculated to fit together the toxicokinetic profiles and the PCB153 measurement at time of the study in 2005–2008 as followed:

- A first simulation of the toxicokinetic profile is done by arbitrarily setting a maximal dose (MD_1_). We thus obtain a PCB153 level estimated at the time of enrollment (PCB153_estimated_)
- The maximal dose in the definitive model (MD_2_) is calculated as follows:
  $${\mathrm{MD}}_{2}=\frac{{\mathrm{MD}}_{1}\times \mathrm{PCB}153_{\mathrm{measured}}}{\mathrm{PCB}153_{\mathrm{estimated}}}$$
  where PCB153_measured_ is the measured level of PCB153 at the time of enrollment.
