# Supplementary material for: Breast Cancer and Exposure to Organochlorines in the CECILE Study: Associations with Plasma Levels Measured at the Time of Diagnosis and Estimated during Adolescence
Source: Int J Environ Res Public Health. 2019 Jan 18;16(2):271. doi: 10.3390/ijerph16020271 (PMC6351946; doi:10.3390/ijerph16020271)
Supplement: Supplementary file 1 [file ijerph-16-00271-s001.pdf]

Supplementary Materials

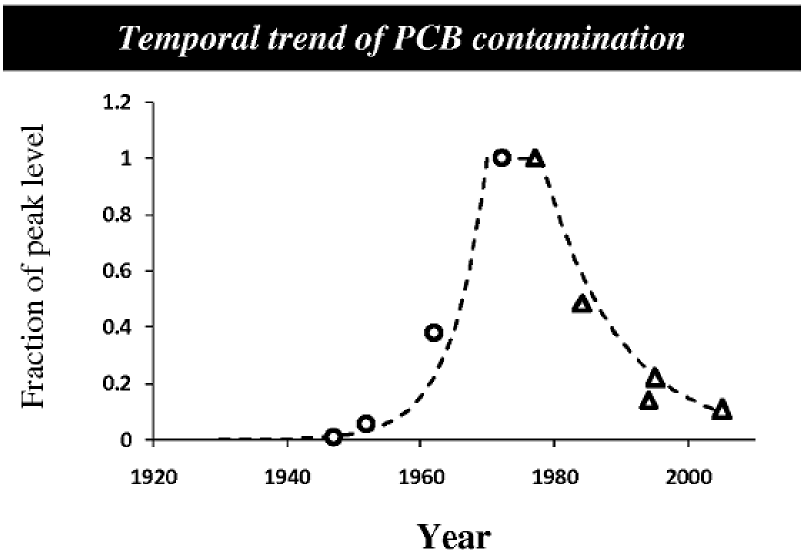

Figure S1. Temporal trend of environmental contamination levels reported as fractions of peak levels based on production data and estimated daily intakes

○ Based on production data [S1]; ▲Based on estimated daily intakes [S2]; Figure taken from [S3].

S1. OCDE. *Rapport sur la Mise en Application par les Pays Membres de la Décision du Conseil sur la Protection de L'environnement par le Contrôle des Diphényles Polychlorés*; OCDE: Paris, France, 1982.

S2. Baars, A.J.; Bakker, M.I.; Baumann, R.A.; Boon, P.E.; Freijer, J.I.; Hoogenboom, L.A.P.; Hoogerbrugge, R.; van Klaveren, J.D.; Liem, A.K.D.; Traag, W.A.; et al. Dioxins, dioxin-like PCBs and non-dioxin-like PCBs in foodstuffs: Occurrence and dietary intake in The Netherlands. *Toxicol. Lett.* **2004**, *151*, 51–61, doi:10.1016/j.toxlet.2004.01.028.

S3. Verner, M.-A.; Bachelet, D.; McDougall, R.; Charbonneau, M.; Guénel, P.; Haddad, S. A case study addressing the reliability of polychlorinated biphenyl levels measured at the time of breast cancer diagnosis in representing early-life exposure. *Cancer Epidemiol. Biomark. Prev. Publ. Am. Assoc. Cancer Res. Cosponsored Am. Soc. Prev. Oncol.* **2011**, *20*, 281–286, doi:10.1158/1055-9965.EPI-10-0992.
